# Human umbilical cord blood-derived mononuclear cells improve murine ventricular function upon intramyocardial delivery in right ventricular chronic pressure overload

**DOI:** 10.1186/s13287-015-0044-y

**Published:** 2015-03-26

**Authors:** Saji Oommen, Satsuki Yamada, Susana Cantero Peral, Katherine A Campbell, Elizabeth S Bruinsma, Andre Terzic, Timothy J Nelson

**Affiliations:** General Internal Medicine and Transplant Center, Mayo Clinic, Rochester, MN USA; Center for Regenerative Medicine, Mayo Clinic, Rochester, MN USA; Department of Molecular Pharmacology and Experimental Therapeutics, Mayo Clinic, Rochester, MN USA; Division of Cardiovascular Diseases, Mayo Clinic, Rochester, MN USA; Autonomous University of Barcelona, Program of Doctorate of Internal Medicine, Barcelona, Spain; Department of Medical Genetics, Mayo Clinic, Rochester, MN USA; Department of Medicine, Mayo Clinic, 200 First Street, SW, Rochester, MN 55905 USA

## Abstract

**Introduction:**

Stem cell therapy has emerged as potential therapeutic strategy for damaged heart muscles. Umbilical cord blood (UCB) cells are the most prevalent stem cell source available, yet have not been fully tested in cardiac regeneration. Herein, studies were performed to evaluate the cardiovascular safety and beneficial effect of mononuclear cells (MNCs) isolated from human umbilical cord blood upon intramyocardial delivery in a murine model of right ventricle (RV) heart failure due to pressure overload.

**Methods:**

UCB-derived MNCs were delivered into the myocardium of a diseased RV cardiac model. Pulmonary artery banding (PAB) was used to produce pressure overload in athymic nude mice that were then injected intramyocardially with UCB-MNCs (0.4 × 10^6 cells/heart). Cardiac functions were then monitored by telemetry, echocardiography, magnetic resonance imaging (MRI) and pathologic analysis of heart samples to determine the ability for cell-based repair.

**Results:**

The cardio-toxicity studies provided evidence that UCB cell transplantation has a safe therapeutic window between 0.4 to 0.8 million cells/heart without altering QT or ST-segments or the morphology of electrocardiograph waves. The PAB cohort demonstrated significant changes in RV chamber dilation and functional defects consistent with severe pressure overload. Using cardiac MRI analysis, UCB-MNC transplantation in the setting of PAB demonstrated an improvement in RV structure and function in this surgical mouse model. The RV volume load in PAB-only mice was 24.09 ± 3.9 compared to 11.05 ± 2.09 in the cell group (mm^3^, *P*-value <0.005). The analysis of pathogenic gene expression (BNP, ANP, Acta1, Myh7) in the cell-transplanted group showed a significant reversal with respect to the diseased PAB mice with a robust increase in cardiac progenitor gene expression such as GATA4, Kdr, Mef2c and Nkx2.5. Histological analysis indicated significant fibrosis in the RV in response to PAB that was reduced following UCB-MNC’s transplantation along with concomitant increased Ki-67 expression and CD31 positive vessels as a marker of angiogenesis within the myocardium.

**Conclusions:**

These findings indicate that human UCB-derived MNCs promote an adaptive regenerative response in the right ventricle upon intramyocardial transplantation in the setting of chronic pressure overload heart failure.

## Introduction

Cell-based therapy has emerged as a potential therapeutic strategy for restoring damaged cardiac tissue with a focus on left ventricular function. There is a spectrum of cell types utilized for cardiac applications including bone marrow-derived mononuclear cells (MNCs) or mesenchymal stromal cells (MSCs) with newer protocols isolating or guiding the expansion of specific subpopulations [1]. Cell-based therapy has offered promising evidence with mixed results that suggest an unharnessed potential for cell therapy that may be tailored for individual needs to impede progressive heart failure [2-4]. Human umbilical cord blood (h-UCB) stem cells have generated significant attention in regenerative medicine with recent studies demonstrating the ability of UCB derived cells to differentiate into various cell types [5,6]. Preclinical studies with UCB cells have demonstrated their efficacy in various diseases, such as heatstroke, amyotrophic lateral sclerosis, post-infarct cardiac regeneration, and liver diseases [4,7-11]. Subsequently, multiple groups have demonstrated that the delivery of UCB cells has the potential to improve cardiac function in animals following acute myocardial infarction (MI) in the left ventricle [12-14]. Recent studies in a novel sheep model of chronic right ventricular volume overload showed that UCB cells transplanted in the right ventricle improved heart function [15]. These studies demonstrated that UCB stem cells are multipotent and capable of differentiating into non-blood cell types [16]. These observations raised the possibility of using autologous UCB cells in congenital disease to repair ventricular myopathy. Furthermore, some of the most refractory forms of congenital heart disease are the result of dysfunctional systemic right ventricle failing in response to chronic pressure overload [17]. Therefore, determining the safe dosing and delivery strategy of UCB-derived cells to promote endogenous regenerative capacity within the right ventricle becomes a critical opportunity for regenerative medicine.

The current studies reported herein were performed to evaluate the cardiovascular safety and efficacy profile of h-UCB-derived MNCs received via intramyocardial delivery into the right ventricle of a pressure overloaded murine model. The murine model with pulmonary artery banding (PAB) restricts the blood flow and causes right ventricular dysfunction due to pressure overload and increased volume of the right ventricle. The present findings indicate that UCB-MNCs may have the capacity to repair the damaged cardiac tissue and will launch further investigations to determine whether UCB-MNC therapy could be used to treat right ventricular heart failure.

## Methods

### Ethical approval

All animal experiments were approved by the ‘Institutional Animal Care and Use Committee’ (IACUC-Protocol A45410), Comparative Medicine, at Mayo Clinic. The experimental animals received care in compliance with the ‘Guide for the Care and Use of Laboratory Animals’. In addition, all experiments were carried out in compliance with the Helsinki Declaration. h-UCB-derived MNCs were isolated from cord blood of normal donors with informed consent according to the institutional guidelines under the approved protocol. The cord blood was collected and processed at Mayo Clinic with the approval of the ‘Mayo Clinic Institutional Review Board’ (IRB protocol- 11–002535) and manufactured to mononuclear cells according to our good manufacturing practice (GMP) process in the Human Cell Therapy Laboratory, Mayo Clinic, Rochester, MN.

### Experimental design

Athymic nude mice at six- to eight-weeks of age were purchased from Harlan Laboratories (Indianapolis, IN, USA). All the animals were housed at the Mayo Clinic animal house facility and maintained under temperature and humidity according to the the guidelines. Mice were housed individually after surgery in polypropylene cages and were allowed water and pelleted food *ad libitum.* Human cord blood was collected from the umbilical cord vein and the mononuclear fraction was isolated from the cord blood by density gradient centrifugation. Subsequently, the obtained cell population was rapidly frozen in CryoStor freezing media, pre-formulated with 10% dimethyl sulfoxide (DMSO). Prior to cell transplantation in the murine heart, the UCB cells were thawed and evaluated for viability.

### Cardiac safety evaluations

**Figure 1:**
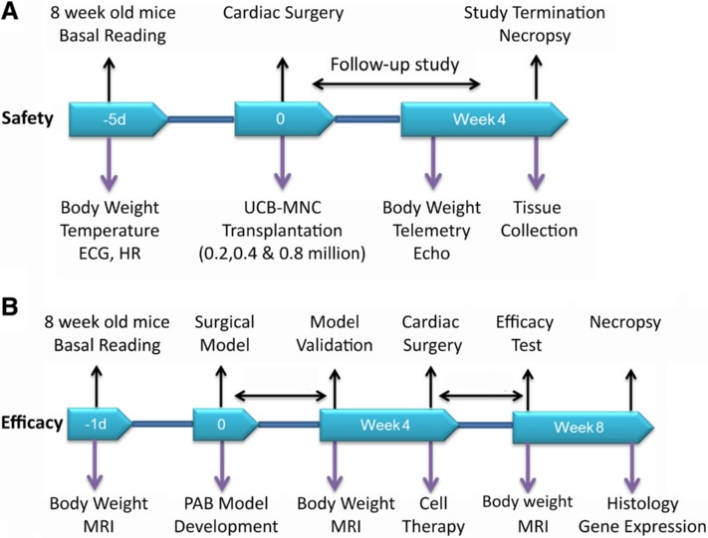
Study design. **(A)** Safety studies: the primary focus of the study was to investigate potential adverse cardiovascular effects of umbilical cord blood mononuclear cells transplantation in the right ventricle of mouse heart. A dose escalation study was carried out to assess the possible side effects and estimate the dosage likely to be the safety margin of UCB-MNCs. **(B)** Efficacy studies: the second part of the study was designed to explore the possible beneficial effects involved in right ventricular remodeling upon intramyocardial injection of UCB cells in a disease model with pressure overload right heart failure. MNCs, mononuclear cells; UCB, umbilical cord blood.

Athymic nude mice were randomly divided into four groups. Prior to telemetry implantation in mice, the baselines of body weight, electrocardiogram (ECG), heart rate, and temperature were monitored (Figure 1A). Subsequently, UCB-MNCs were transplanted in the myocardium of the right ventricle. Cardiovascular safety parameters were monitored for three weeks after cell transplant and the animals were then sacrificed for gross and histopathology.

### Surgical procedure for DSI transmitter implantation

Animals were subcutaneously implanted with a telemetry device (PhysioTel and TA ETA-10, Data Science International, St. Paul, MN, USA) for remote and long-term monitoring of physiological and bioelectrical variables (for example, blood pressure, heart rate, ECG) in conscious, unrestrained animals. On the day of the experiment, the animals were weighed and anesthetized for telemetry transmitter implantation according to the animal protocol approved by IACUC (Institutional Animal Care and Use Committee). During surgery, animals were maintained in a surgical plane of anesthesia, on a heating pad with close monitoring of vital signs and ECG. A small telemetric transmitter devise was implanted in the ventral abdominal area subcutaneously under isoflurane anesthesia. The paired wire electrodes (negative and positive leads) were placed under the skin of the thorax. The skin incision was closed by sutures and animals were used for cell transplantation three to seven days after surgery.

### Telemetry data acquisition and recording

ECG and temperature were recorded continuously for three hours prior to UCB cell injections with DSI data acquisition software. The ECG was monitored for three hours continuously after cell injection. The ECG, temperature and other parameters such as body weight were recorded every three days up to three weeks. The ECG waveform was displayed and recorded by Dataquest software and analyzed to determine time latency for QT intervals and heart rate changes by DSI Ponemah software.

### Umbilical cord blood-derived MNCs transplantation to the right ventricle

Mice were anesthetized in a closed chamber filled with oxygen and 2% to 3% isoflurane. The chest was shaved and mice were placed on a heating pad at a temperature of 37°C. The mice were intubated with a 20G needle catheter and mechanically ventilated with a Harvard mini-ventilator (model 687, Hugo Sacks, Elektronik, Germany) at a rate of 180 breaths per min and a tidal volume of 125 μl. Following ventilation, an incision was made through the 4th and 5th right intercostal space to expose the epicardium of the right heart. The cell treated groups of mice received an intramyocardial injection (0.2, 0.4 or 0.8 × 10^6^ cells/mouse) to the right ventricle (five injections of 2.5 μl each) by using a syringe pump 11 elite (Harvard Apparatus, Holliston, MA, USA).

### Echocardiography

Three weeks following cell transplantation, right/left ventricular function and structures, and the absence of adverse effects, including uncontrolled growth were prospectively evaluated by echocardiography (Vevo2100 with a MS-400 30-MHz transducer, Visual Sonics, Toronto, Canada). The animals were sedated with isoflurane inhalation (2%) and long and short axis views were obtained with both M-mode and two-dimensional echo images.

### Efficacy evaluations

#### Right ventricular pressure overload via pulmonary artery banding

Athymic nude mice six- to eight-weeks of age underwent pulmonary artery banding (Figure 1B). A left lateral thoracotomy was performed through the 2nd intercostal space and exposed the heart [18]. After mobilization of the pericardium, the pulmonary trunk was bluntly dissected with a curved fine forceps from the aorta and left atrium. A tunnel was created underneath the pulmonary trunk using an L-shaped 28 gauge blunted needle. Following this procedure, a 7–0 surgical suture was placed around the pulmonary artery and tied over a 25G needle bent into an L-shape. The L-shaped needle was removed immediately to leave a consistent ligature around the pulmonary artery. The chest cavity was closed layer by layer. As the growth of the animals increases, the fixed diameter ligature results in a pressure overload in the right ventricle. Mice that did not receive any treatment (that is, surgery and cells) served as controls.

### Pulmonary artery banding and cell implantation into right ventricular myocardium

Four weeks after PAB and model validation, another thoracotomy was performed as previously described for the purpose of delivering the cells directly into the epicardium of the right ventricle. The UCB-MNCs (0.4 million cells/heart) were injected into four or five sites of the right ventricle as previously described with each injection being no more than 2.5 μl.

### Cardiac magnetic resonance imaging

Cardiac magnetic resonance imaging (MRI) was performed both four weeks after PAB and four weeks after cell transplantation by magnetic cardiac imaging (16.4 T Bruker ultrashield 700WB plus scanner, Bruker BioSpin Corporation, Billerica, MA, USA). The cardiac MRI performed prior to PAB was considered a control (basal) recording, followed by banding (after four weeks) and then cell transplantation with another four weeks of monitoring. The mice were anesthetized and placed in a cone shaped chamber connected to a ventilator for the entirety of the scanning procedure. The scan was adjusted by heart position and by different modes of scan, such as transversal, coronal and sagittal [19]. The scanning was done in short axis and a stack of seven 1 mm slices was measured in the base area of the right ventricle (RV). The acquired MRI data were analyzed with digital imaging software (‘Analyze,’ Biomedical Imaging Resource, BIR, Mayo Clinic, Rochester, MN, USA). Each DICOM series was loaded into a three-dimensional volume. Thresholding was used in each image of the dataset to segment the blood pool, with contrast, in the atria. Voxel counting and voxel dimensions were used to determine the volume.

### Real-time PCR analyses

Real-time RT-PCR analysis was carried out using RNA isolated from the heart tissue (free wall RV) using an RNeasy Plus Mini kit (Qiagen, Valencia, CA, USA). The reverse transcriptase (RT) reaction was performed using an iScript cDNA synthesis kit (Bio-Rad, Hercules, CA, USA). Quantitative assessment of predictive cardiac markers, ANP, BNP, Acta-1, Myh7, Myh6, and cardiac progenitor markers, GATA-4, Kdr, Mef2c and Nkx2.5, was performed by real-time PCR. The target values were normalized to GAPDH and are expressed as 2^−ΔΔCt^ (fold difference).

### Immunofluorescence

The expression of Ki-67 and CD31 staining for blood vessel formation was determined in PAB and cell treated ventricles by immunofluorescence staining and observed via confocal microscopy. The primary antibodies used for the immunolabeling studies were rabbit polyclonal to Ki-67 and CD31 (Abcam, Cambridge, MA, USA), fixed paraffin sections at 1:200 for identification of Ki-67 positive cells and endothelial cells, respectively. The samples were then incubated with the secondary antibody, anti-mouse FITC-conjugated, for one hour. The immunofluorescence staining was acquired with a Zeiss LSM 510 confocal microscope. The quantification of Ki-67 and CD31-positive blood vessels was done by CellSens Dimension software.

### Tissue collection and histopathology

Hearts were harvested from control and experimental groups of animals at four and eight weeks after cell transplantation. For histological examination, hearts were fixed in 10% neutral formalin and embedded into paraffin by standard techniques. After serial sectioning of hearts (apex to base), 5-micron sections were stained with hematoxylin and eosin (H & E) and Masson Trichrome stain for pathological observations. H & E sections were examined by light microscopy for any tumor formation and fibrotic areas were observed by analysis of Masson Trichrome stained sections. The heart tissue was excised and the free walls of the RV and left ventricle (LV) were separated and stored in −80°C for RNA isolation.

### Statistical analysis

Data are presented as mean ± SE of the mean of at least four independent experiments. Statistical analysis was performed using Graph Pad Prism 5 (La Jolla, CA, USA) software. The differences between the groups were evaluated by unpaired one-way analysis of variance (ANOVA) test and differences were considered significant when a *P*-value <0.05.

## Results

### Safety of intramyocardial transplantation of umbilical cord blood-derived mononuclear cells in the right ventricle

**Figure 2:**
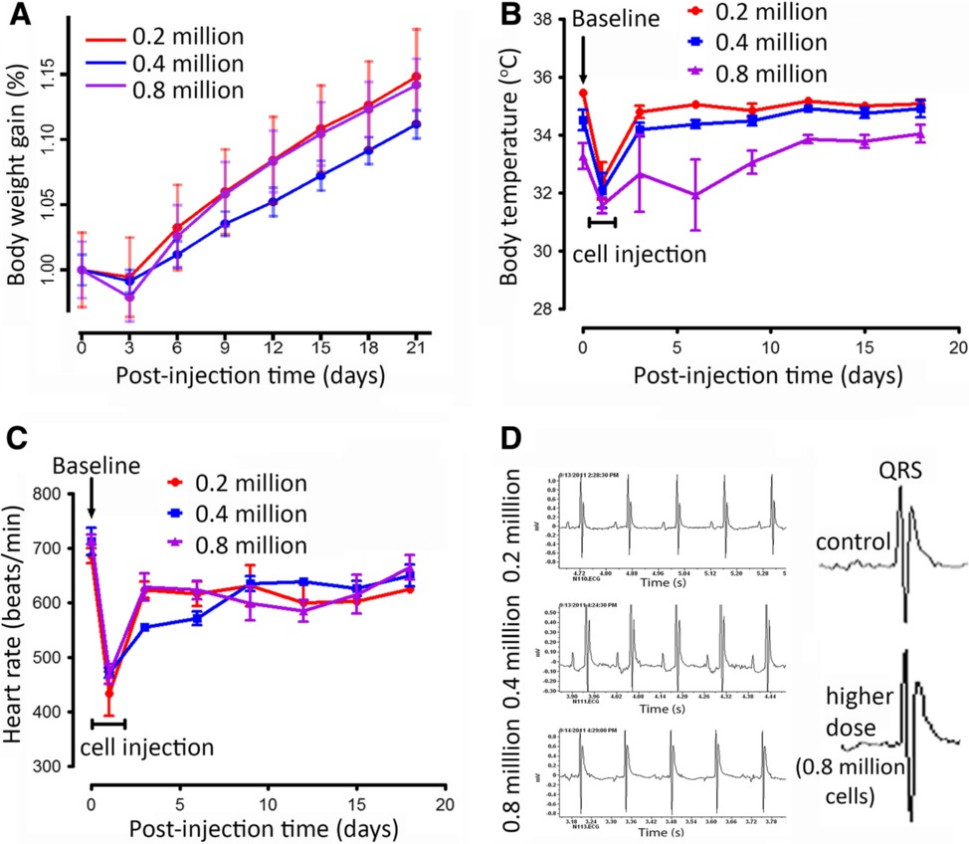
Lack of evidence for ventricular arrhythmias and QT interval prolongation after UCB-MNCs transplantation. Multiple doses (0.2, 0.4 and 0.8 million cells) of UCB-MNCs were transplanted on the right ventricle to evaluate cardiac safety and tolerability (n = 7). **(A, B)** Body weights and temperatures measured up to three weeks followed by UCB-MNCs transplantation. Heart rate (HR) was derived from telemetric recording in conscious animals **(C)**. Data presented as means ± SE. **(D**) ECG tracing from a conscious mouse implanted subcutaneously with a telemetry transmitter recorded at baseline and 3, 6, 9, 12, 15, 18 and 21 days after intramyocardial injection of UCB-MNCs. UCB-MNCs, umbilical cord blood-mononuclear cells.

*In vivo* cardiovascular tolerance studies using three doses (lower, mid and higher) of UCB-MNCs administered into the myocardium of normal mice demonstrated the safety dose for the RV. Animal body weights were monitored before and after cell injections. There was no weight loss within three weeks of cell transplantation (Figure 2A). Furthermore, body temperature was used as a marker of overall health of the animal and any allergic or infectious side effects and was monitored daily following the cell transplantation. Following injection of UCB cells, a decrease in body temperature was noticed on day 1; however, by three to five hours post-surgery the body temperature returned to normal (Figure 2B). Telemetry devices were implanted and recorded the baselines of HR and ECG prior to cell delivery. The UCB-MNCs were then transplanted and animals were followed for three additional weeks. The telemetry implantation, cell transplantation, and recovery periods were well tolerated in all mice upon standardization of each protocol. The data recordings were generated for three hours at each time point and were analyzed by DSI Ponemah software. The mouse baseline HR prior to cell injection is approximately 600 to 700 beats/minute (Figure 2C). A lower HR was observed on day 1 due to surgical manipulation, but thereafter there was no significant difference in heart rates between control and cell transplanted groups from baseline recordings. Furthermore, UCB-MNCs did not alter the QT-segments or the morphology of ECG waves after cell transplantation at doses of 0.2, 0.4 or 0.8 million cells/mouse (Figure 2D). The doses used here demonstrated an acceptable safety margin for h-UCB cells to be administered intramyocardially.

### Ventricular structure and function following intramyocardial delivery of UCB-MNCs

**Figure 3:**
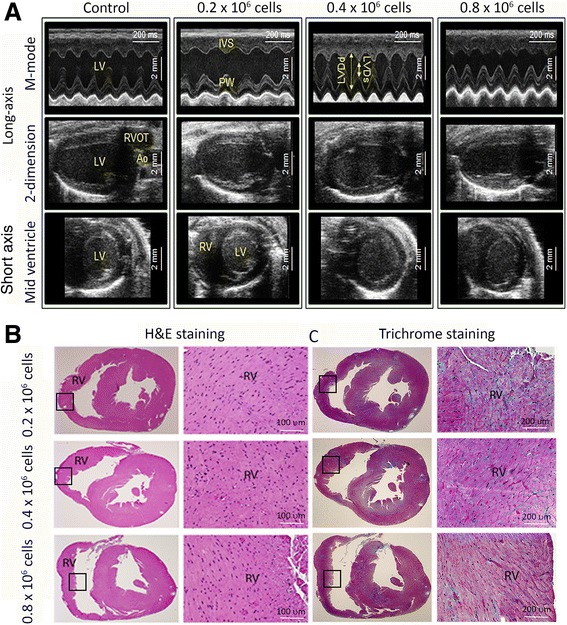
No detectable risk of cardiovascular toxicity from UCB-MNCs delivered into the myocardium of the right ventricle. **(A)** Echocardiographic images three weeks after UCB-MNC transplantation indicating normal cardiac function and no signs of tumor formation in any of the doses. A comparison (0.2 million (low dose) to 0.8 million cells (high dose)) of echocardiography was performed in this study. **(B)** Histo-pathological sections stained with hematoxylin & eosin (H & E) three weeks after RV myocardial injection. Short-axis sections were used to identify the area of injection. Higher magnification images were captured at x100. UCB-MNC transplantations showed no incidence of lesion in the RV and no signs of tumor formation upon histological examination in any animals. **(C)** Heart sections stained with Masson Trichrome stain (blue) after myocardial injection of UCB-MNCs. The average ratio of fibrosis area per RV area was measured to determine the fibrotic area. N = 4, Scale bars = 200 μm. RV, right ventricle; UCB-MNCs, umbilical cord blood-mononuclear cells.

Low to high doses of UCB-MNCs delivered to the RV were followed by echocardiography after three weeks of cell transplantation. Structure and function was equivalent between controls and mice administered UCB-derived MNCs at all doses. Notably, echocardiography showed normal left and right ventricular size/function and no tumor mass was observed (Figure 3A). The data demonstrated normal sinus rhythm and showed no evidence of any tumor or abnormal mass effect in control or cell treated groups of animals. The left ventricular function was preserved at the end of this study period. Three weeks of follow-up study reflected no significant difference in diastolic and systolic volume within both groups of animals. Cardiac tissue of both control and cell transplanted mice was harvested three weeks after cell delivery for histological evaluation. Myocardial tissue was stained with H & E and examined for any tumor formation. Histological data evaluated by light microscopy did not show any evidence of lesions or tumor growth in the experimental group or control group (Figure 3B). Masson Trichrome staining did not reveal any fibrotic area in either group of animals (Figure 3C). Overall, the cardio-toxicity studies provided evidence that cell transplantation has a safe therapeutic window between 0.4 and 0.8 × 10^6^ cells/heart. This data demonstrated no detectable risk for cardiovascular toxicity of UCB-MNCs delivered into the myocardium of the right ventricle.

### Efficacy of UCB-derived MNCs transplanted into chronic RV pressure overload

#### Evaluation of structural changes in the pressure overloaded right heart

**Figure 4:**
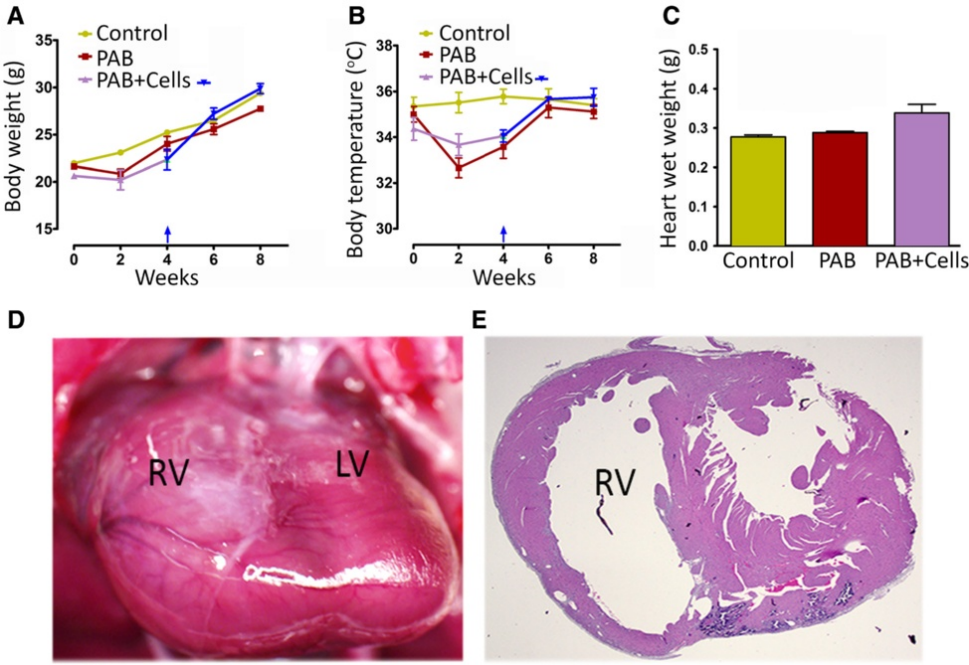
Chronic pressure overload results in morphological and functional changes in the right ventricle**.** Four weeks after pulmonary artery banding, UCB-MNCs were transplanted into the right ventricle. Body weights and temperatures were recorded at baseline and two, four, six and eight weeks **(A, B)**. There was no significant difference in weight gain between the control, PAB-only and PAB + cells groups. The data depict six animals in each group. **(C)** Wet weight of whole heart was recorded eight weeks after pulmonary artery banding. A comparison was made between cell transplanted and PAB-only groups. A trend towards increase in heart weight was observed in the cell transplanted group (n = 6; *P* >0.5). **(D**) The PAB model was validated prior to cell delivery. After PAB, the right ventricle is exposed to pressure overload by pulmonary outflow tract stenosis resulting in right ventricular dilation **(E)**. PAB, pulmonary artery banding; UCB-MNCs, umbilical cord blood-mononuclear cells.

A surgical PAB model with severe RV pressure and increased volume was created in immunodeficient mice to allow for safety and efficacy studies of h-UCB-derived cells. The average body weight and temperature was marginally decreased between one and two weeks of surgery; however, there was no significant difference in weight gain after four and eight weeks of follow-up (Figure 4A). Significant changes in the body temperature observed between one and four week after surgery (Figure 4B). MNCs administration in the RV showed a marginal increase in heart wet weight; however, the PAB only group did not show any significant increase in heart weight at eight weeks of the study period (Figure 4C). Mice with a pressure overloaded RV demonstrated RV dilation after four weeks as shown by an increased volume in the right ventricular chamber (Figure 4 D, E).

#### UCB-derived MNC therapy on right ventricular remodeling after chronic right ventricular pressure overload

**Figure 5:**
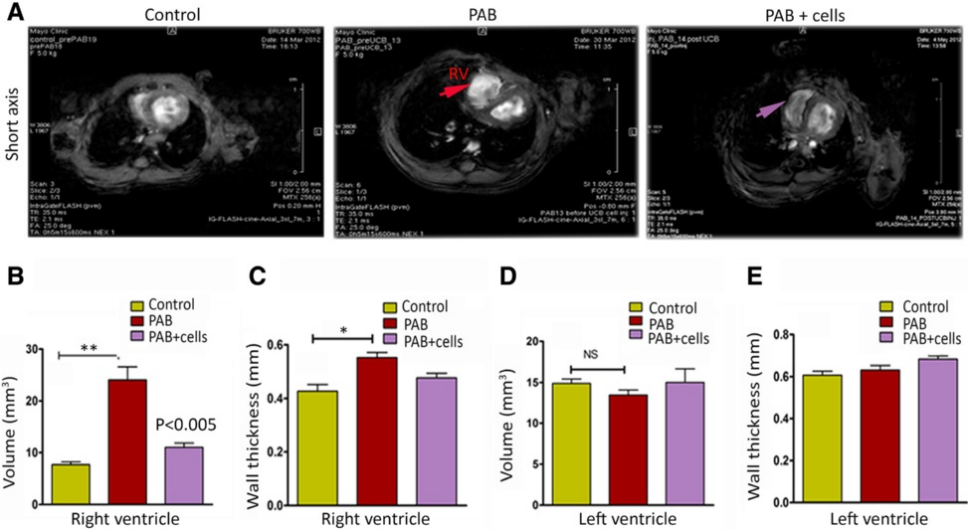
Myocardial delivery of UCB-MNCs improves RV function and favorable RV remodeling after pulmonary artery banding. **(A)** Short-axis image obtained from magnetic resonance imaging in the three experimental groups. Pulmonary artery banding (n = 6) produced severe RV dilation and ventricular dysfunction when compared with control (sham) animals (n = 6). Four weeks after cell transplantation, there was a less-pronounced RV dilation in the UCB-MNCs transplanted group (n = 6). **(B)** Magnetic resonance imaging-derived right ventricular volumes in control, PAB and cell transplanted group. The PAB only group demonstrated an increase in RV volume (** *P*-value <0.005 versus control). Intramyocardial delivery of UCB-MNCs indicated a reduction in RV volume (*P*-value <0.005 versus PAB). **(C)** Right ventricular wall thickness was compared among groups; the PAB-only animals showed a significant increase in RV wall thickness (* *P*-value <0.05); however, the cell transplanted group demonstrated a smaller reduction of the RV wall thickness. **(D, E)** No significant changes in LV functions were noticed between the experimental groups. LV, left ventricle; PAB, pulmonary artery banding; RV, right ventricle; UCB-MNCs, umbilical cord blood-mononuclear cells.

The second aim of the study was to examine whether UCB-derived MNC injection in the pressure overloaded RV would enhance cardiac function compared to the PAB-only. After the PAB model validation by MRI, 0.4 million cells were injected into the right ventricular myocardium to measure the recovery of RV function. Average body weight and temperature throughout the eight-week period of study indicated a marginal weight gain in the cell treated group of animals (Figure 4 A, B). After four weeks of cell treatment, MRI was used to monitor changes in cardiac function and structure. The baseline MRI studies were performed prior to PAB surgery. The dysfunctional RV model was validated in MRI studies prior to UCB-MNCs delivery. Pulmonary artery constriction caused a failing heart phenotype including RV chamber dilation and right ventricular dysfunction (Figure 5A). Four weeks after PAB, a severe RV dilation was noticed in all animals (Figure 5-top panel). Four weeks after UCB-MNCs injection, MRI revealed a recovery of right ventricular chamber size and volume. The right ventricular volumes were measured from multi-section images (ventricular short axis). The RV volume loading PAB mice was 24.09 ± 3.9 compared to 11.05 ± 2.09 in the cell treated group (mm^3^; *P* = 0.005) (Figure 5B). Imaging analysis showed an increase in wall thickness and reduced RV chamber size in the cell treated group compared to PAB only experiments (Figure 5C). There was no significant change in LV functions (Figure 5D, E).

#### UCB–derived MNC therapy suppressed the PAB-induced increase in pathogenic gene expression

**Figure 6:**
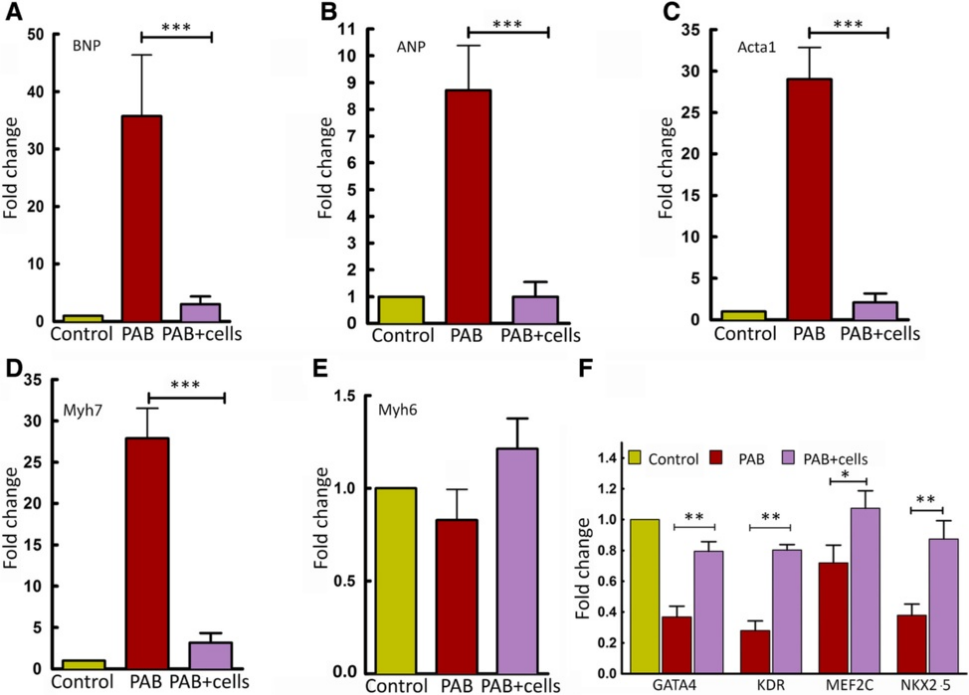
UCB mononuclear cells suppressed PAB-induced increase in pathogenic gene expression and upregulated cardiac transcription factors. **(A through E)** Right ventricular free wall was used for quantitative real-time PCR analysis of cardiac pathogenic markers, such as BNP, ANP, Acta1, Myh7 and Myh6. Transcript levels of these markers were measured in heart tissue of the PAB-only group and compared with that of the UCB-MNC transplanted group. **(F)** Cardiac transcription factors (GATA4, Kdr, Mef2c and Nkx2.5) were significantly upregulated in the UCB-MNC transplanted heart tissue in comparison with PAB-only heart tissue (n = 4, ** *P*-value <0.05 versus PAB). Data are expressed as fold change. All data reported are representative of four independent experiments performed in triplicate and are normalized to those of GAPDH. Data are presented as mean ± SEM. PAB, pulmonary artery banding; SEM, standard error of the mean; UCB-MNCs, umbilical cord blood-mononuclear cells.

Specific cardiac pathogenic markers were used for the diagnosis of heart failure and recovery. The dysfunctional heart tissue of PAB mice showed an elevated level of pathogenic gene expression (BNP, ANP, Acta1and Myh7) compared to a significant reversal of these genes in the group of animals receiving cell transplantation (Figure 6A- D). The expression of Myh6 was not significantly changed under these tested conditions (Figure 6E). Transcriptional regulation by multiple cardiac transcription factors, such as GATA4, Kdr, Mef2c, and Nkx2.5, is associated with an adaptive cardiac regenerative process [20]. After PAB, the transcript levels of cardiac progenitors GATA4, Kdr, Mef2c, and Nkx2.5 were significantly lower than those of control tissue. The embryonic gene expression profile was reversed upon transplantation of UCB-MNCs (Figure 6F).

### UCB-derived MNCs reduce the fibrosis of PAB-induced ventricular injury and promote proliferation, angiogenesis

**Figure 7:**
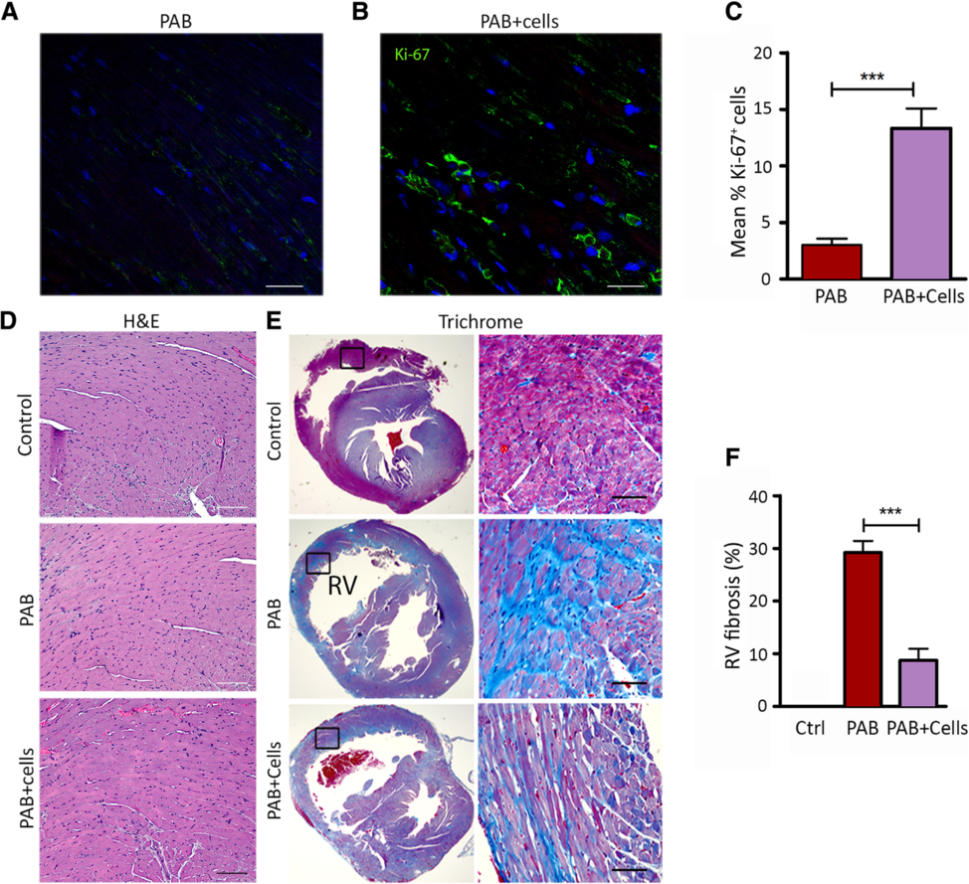
UCB-MNCs transplantation augments myocardial cell proliferation and attenuates myocardial fibrosis**.** Myocyte proliferation was assessed by Ki-67 immuno-fluorescence staining in RV of **(A)** PAB-only and **(B)** UCB-MNCs treated animals. Ki-67 positive cells were stained green and nuclei were in blue. The Ki-67 expressions were quantified and data are shown in a bar graph **(C**). Cell transplantation of UCB-MNCs results in enhanced cellular proliferations when compared to PAB-only animals (***P–value <0.005 versus PAB; scale bars = 100 μm). The images were representatives of three PAB-only and three cells treated samples. **(D**) Representative images of control, PAB and UCB-MNCs transplanted RV (n = 5 each) stained for hematoxylin and eosin (H & E) for any lesions or tumor formation. **(E)** Histological sections were stained with Masson-Trichrome stain (blue) to identify the fibrotic area of RV in response to PAB and cells delivery. Viable myocardial cells was stained red and fibrosis was blue (scale bars = 50 μm). **(F)** Percentage circumferential fibrosis measured in short-axis Masson-Trichrome staining was significantly smaller in UCB-MNCs transplanted heart compared to the PAB-only group (***P–value <0.005 versus PAB and control). PAB, pulmonary artery banding; RV, right ventricle; UCB-MNCs, umbilical cord blood-mononuclear cells.

**Figure 8:**
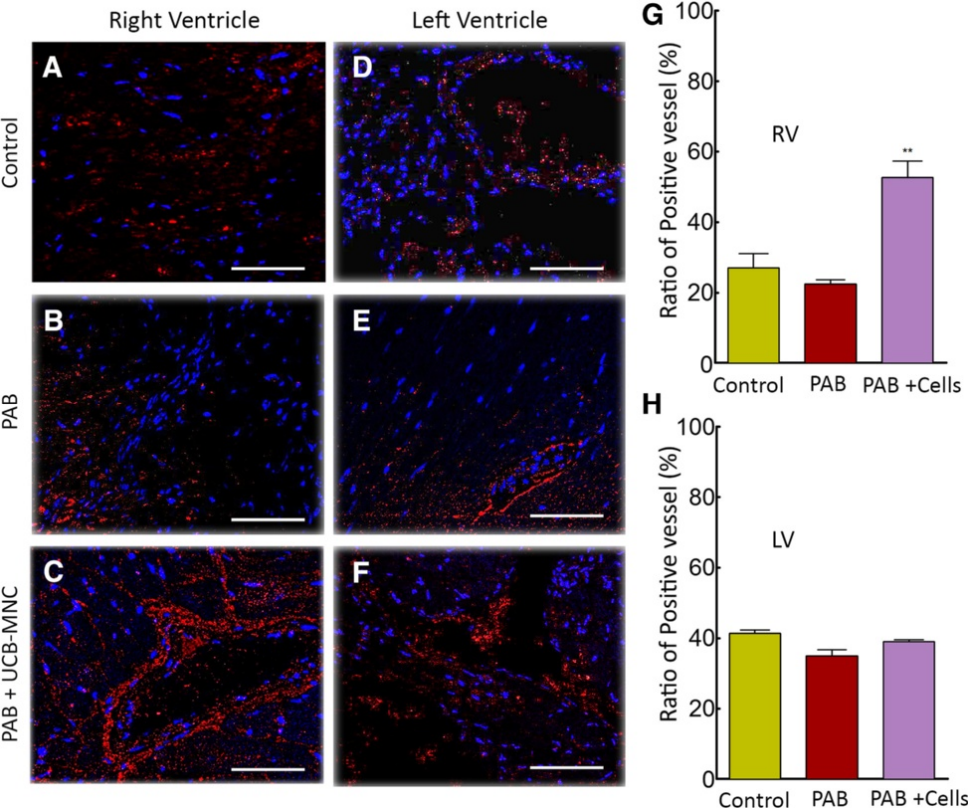
Mononuclear cells of UCB paracrine action and vascular reactivity in right ventricle. Immunofluorescence analysis for the angiogenesis marker CD31 was performed on paraffin embedded cell-treated and PAB only heart tissue. **(A-F)** Vascularity in RV and LV was assessed, and resulted in an increase in the number of blood vessels in RV after four weeks of cells delivery (*P* = 0.01 versus PAB, scale bars = 100 μm). **(G, H)** CD31 positive vessels (endothelial-red) were analyzed through CellSens Dimension software (n = 4) and non-specific/background was filtered out for final calculation. A representative image (LV, RV) of immunofluorescence staining is illustrated. LV, left ventricle; PAB, pulmonary artery banding; RV, right ventricle; UCB, unbilical cord blood.

Animals were sacrificed after the eight-week follow-up study and heart tissue was collected for immunofluorescence and histopathology. Cardiomyocyte proliferation in the RV was examined by immunofluorescence using a Ki-67 antibody. The nuclear protein Ki-67 expression occurs in all phases of the cell cycle [21]. Images of heart tissue from PAB with cell transplantation revealed an increase in expression of Ki-67 positive cells in the regions adjacent to fibrosis (Figure 7A, B). Ki-67 expressing cells increased significantly from 3% ± 1% in the PAB only group to 13.33% ± 3.05% after UCB-MNCs injection (*P*–value <0.005, Figure 7C). H & E staining did not show any lesions or tumor formation in any of these animal groups (Figure 7D). Mason-Trichrome stain indicated significant fibrosis formation in the RV area of mice in response to PAB (Figure 7E). The blue color areas were analyzed using cellSens dimension software. Animals that received UCB-MNCs demonstrated minimal fibrosis (8.75% ± 4.3%) compared to PAB-only animal groups (29.25% ± 4.34%), with a significant difference (*P*-value <0.005) between these two groups (Figure 7F). Thus, the UCB cell delivery in the setting of PAB demonstrated a significant reduction in RV fibrosis compared to the PAB-only group. Immunofluorescence staining revealed a significant increase (approximately 50%) in CD31 expression in the RV after UCB-MNC transplantation compared with the PAB-only group of animals. The small and large blood vessels (endothelial) demonstrated a significantly higher percentage in the myocardium (Figure 8; *P* = 0.01) of RV, whereas, the blood vessel density in the untreated LV demonstrated no detectable difference between the cells group and the PAB-only group.

## Discussion

UCB offers a prevalent cell source for autologous regenerative applications in the setting of *in utero* diagnosis such as congenital heart disease. The collection and processing of UCB has been standardized for hematopoietic stem cell transplantation with optimization now required for emerging downstream applications. With a focus on right ventricular dysfunction due to pressure overload, we herein empirically tested the safety and efficacy of h-UCB derived MNCs within an immunodeficient physiological system. Using real-time monitoring systems, the safety of the direct intramyocardial injection of h-UCB-derived MNCs into the RV of a murine model system was determined in a dose–response study. Furthermore, this study established the efficacy of UCB-derived MNCs in preventing maladaptive right ventricular remodeling leading to end-stage heart failure indicated by biochemical and histological evidence of disease. Collectively, h-UCB can be processed to produce a point-of-care product for intra-cardiac delivery that has a wide range of safe doses and is capable of reversing the pathological changes associated with chronic pressure overload on the RV.

The current study is notable for the rational dose selection of h-UCB-derived MNCs and safe delivery to the right myocardium. UCB stem cells have been delivered to the myocardium safely in animals and also have been used in clinical trials for the treatment of myocardial infarction [12,22-24]. The major safety concern of using stem cells to repair the diseased heart is the occurrence of arrhythmias and inflammatory tissue damage [25]. The first part of our study was focused on safety assessment and dose selection of UCB cells to be delivered to a murine model with RV pressure overload. The long-term safety study determined the risk-to-benefit ratio of UCB-MNCs transplantation to the RV of the mouse heart using real-time monitoring telemetry systems. This study demonstrated that the manufactured UCB-derived product did not show any arrhythmic incidence following injection into the RV. Specifically, the UCB-MNCs did not alter QT or ST-segments or the morphology of ECG waves after cell implantation at 0.2, 0.4 or 0.8 million cells/heart. Notably, ECG showed that all animals receiving cells maintained a normal rhythm, ventricular size and function with no evidence of tumor formation. These results are consistent with previous studies using bone marrow that reported no incidence of ventricular arrhythmias and inflammation in humans [26]. Overall, the cardio-toxicity studies provide evidence that UCB-MNCs transplantation has a safe therapeutic window up to 0.4 to 0.8 × 10^6^ cells/heart in normal physiological systems.

Appropriate heart failure model systems with diseased myocardium are an important component for advanced product development of novel regenerative medicine. Several models have been developed recently for cardiac complications, such as myocardial infarction, and address the heart dysfunctions by using a cell therapy method. RV remodeling comprises multiple adaptation mechanisms to increased pressure volume overload, which in concert determine RV performance as well as clinical outcome in patients [27]. MRI findings indicate that a mouse model at four weeks post-PAB has a dilated RV chamber with increased pressure load. As the ventricular contractile weakening progresses, it causes diminished cardiac output that results in a significant RV hypertrophic response [28,29]. Similar results were observed in MRI studies in patients with chronic pressure overload [30,31]. The data attained from the RV pressure overload are in line with earlier reports [19,27,32]. In patients with congenital heart disease, the RV is subjected to abnormal loading conditions and this dysfunction is a major cause of mortality [33]. These observations are consistent with our mouse data and support the physiological relevance of this RV heart failure model system.

UCB cells have emerged as a viable alternative to other sources of stem cells and are most widely used in hematopoietic stem cell therapy [34,35]. UCB is a rich source of hematopoietic stem/progenitor cells with enhanced potency of myogenic differentiation and proliferative characteristics. In addition, UCB could be used in clinical settings for cell transplantations [36-38]. To assess the efficacy in a more clinically relevant cardiac model, UCB-MNCs were delivered into the myocardium of mice with severe right ventricular failure. We have examined the data of ventricular performance, remodeling of the heart, pathology and gene expression analysis four weeks after cell injection. After four weeks, cell injections to the injured RV showed benefit based on RV pressure overload, morphology and structural aspects compared with the PAB-only mice. Imaging analysis showed an increase in wall thickness and reduced chamber size of RV in the cell group compared to PAB group animals. Several studies have shown that UCB cell transplantation increased neovascularization and thus improved cardiac function [39,40]. A recent study also observed that the LV exhibited an improved function after cell transplantation, suggesting a paracrine effect to improve cardiac function [41]. The present data demonstrated that UCB cells are capable of enhancing myocyte proliferation and improving cardiac function; however, the underlying mechanisms remain not fully understood. Our data provide another example of paracrine mechanism of UCB-MNCs that could be coupled with angiogenesis to contribute towards cardiac repair, supported by substantial evidence highlighting the importance of the paracrine effect of stem cells [42]. We demonstrate here that UCB-MNC injection to the RV after four weeks of PAB is sufficient to initiate myocardial angiogenesis. Since we observed an increased endothelial marker (CD31) expression in the RV after cells injection, these data suggest that a paracrine mechanism of UCB-MNC stimulated angiogenesis. These observations demonstrating the paracrine mechanism of UCB cells that play a role in tissue regeneration and stimulating angiogenesis were confirmed [43]. The improved heart functions in PAB mice following cell injection suggest that paracrine effects may be playing a vital role in RV remodeling as well.

Beyond the pathophysiology response at the organ level due to PAB, the cellular response to pressure overload in the RV has not been fully elucidated in a murine model system. The pressure overloaded PAB model and our pathology data demonstrate that the phenotype recapitulates biochemical and cellular changes consistent with heart failure. In the present study, we measured specific cardiac gene expression, such as ANP, BNP, acta-1 and Myh-7, in the RV of a pressure overload murine model in response to intramyocardial UCB-MNCs delivery. Significantly higher expression levels of these sensitive markers seen in the PAB-only group correlate with the severity and prognosis of heart failure [44,45]. Cell transplantation in the myocardium of banded animals showed a significant reversal of these cardiac predictive markers. These results indicate that UCB-MNCs delivery into the myocardium prevents the over expression of cardiac hypertrophy markers ANP, BNP, Acta-1 and Myh7 in the RV. The upregulation of Myh7 and downregulation of Myh6 are common in human heart disease [46]. The ratios of expression levels of Myh6 and Myh7 after UCB-MNCs delivery to the RV have shown beneficial effects on cardiac contractility. Collectively, these data reveal that UCB-MNCs improve myocardial energetics in cardiac hypertrophy and preserve the heart function after RV pressure overload. Previous studies demonstrate that cardiac progenitors play a vital role in pathological remodeling of the RV [47,48]. The fibrosis formation and failing heart phenotype including RV chamber dilation in the PAB-only group profoundly blunted the gene expression of GATA-4, kdr, Mef2c and Nkx2.5. The up-regulation of these cardiac markers may indicate the stimulation of endogenous cardiac progenitors after cells transplantation in the myocardium. The endogenous cardiac progenitor stimulation could diminish pathological remodeling and improve cardiac function.

Cardiac fibrosis is a common feature in patients with severe cardiac pathology and leads to secondary complications with disruption of electro-mechanical performance [49]. The right ventricular pressure overload hypertrophy in the PAB-only group of mice was accompanied by extensive fibrosis in the ventricular wall with a significant reversal upon treatment with UCB-derived mononuclear cell therapy. The current study indicates that UCB-MNCs may have dramatic anti-fibrotic properties and enhance cardiac repair for normal cardiac function. A possible explanation for UCB cells to decrease fibrosis in the RV wall is correlated with the paracrine effect and proliferation of endogenous cells in the heart. The vascular remodeling after cell injection reduces collagen content and thus changes the extracellular matrix. Diminished expression of Ki-67 in PAB-only animals is in line with results reported in patients with heart disease due to aortic valve stenosis who have lower Ki-67 expression [50]. Immunofluorescence images of UCB-MNC treated RV sections stained with Ki-67 indicated proliferating cells in the fibrotic area with preserved myocardial architecture. Although these studies are limited to a single marker with tissue-based analysis, these data indicate a powerful response to cell transplantation. Therefore, the paracrine effect of UCB-derived cell-based therapy may be directly stimulating endogenous cellular proliferation to avoid fibrotic deposition and pathological changes in the RV. These observations are consistent with recent reports that in response to tissue injury, UCB cells can express and secrete paracrine factors that can activate endogenous repair mechanism [51].

## Conclusions

Congenital heart disease leads to significant long-term morbidity due to inherently compromised cardiac structure and function. Regenerative medicine offers a promising outlook to rebuild the myocardium if cell-based therapies can be safely achieved with feasible protocols. Herein, h-UCB-derived MNCs have been collected, processed, and monitored within a murine model system for cardiac toxicity. The results indicate that a wide therapeutic window exists for UCB-derived cells with direct intramyocardial delivery and the target dose of 0.4 million cells/animal demonstrated reversal of pathological changes in the RV due to pressure overload heart failure. These results provide the rational design for future studies applying autologous regenerative strategies for congenital heart disease limited by right ventricular performance.

## Abbreviations

Ao: aorta
ECG: electrocardiogram
H & E: hematoxylin and eosin
HR: heart rate
IVS: interventricular septum
LV: left ventricle
LVDd/LVDs: left ventricular diastolic/systolic dimension
MNC: mononuclear cell
MRI: magnetic resonance imaging
PAB: pulmonary artery banding
PW: posterior wall
RV: right ventricle
RVOT: right ventricular outflow tract
UCB: umbilical cord blood
